# Designer biomass for next-generation biorefineries: leveraging recent insights into xylan structure and biosynthesis

**DOI:** 10.1186/s13068-017-0973-z

**Published:** 2017-11-30

**Authors:** Peter J. Smith, Hsin-Tzu Wang, William S. York, Maria J. Peña, Breeanna R. Urbanowicz

**Affiliations:** 1 Complex Carbohydrate Research Center, University of Georgia, 315 Riverbend Road, Athens, GA USA; 2 BioEnergy Science Center, Oak Ridge National Lab Laboratory, Oak Ridge, TN USA

**Keywords:** Xylan, Glucuronoxylan, Arabinoxylan, Biosynthesis, Recalcitrance, Polysaccharide, Cell wall, Bioindustry

## Abstract

Xylans are the most abundant noncellulosic polysaccharides in lignified secondary cell walls of woody dicots and in both primary and secondary cell walls of grasses. These polysaccharides, which comprise 20–35% of terrestrial biomass, present major challenges for the efficient microbial bioconversion of lignocellulosic feedstocks to fuels and other value-added products. Xylans play a significant role in the recalcitrance of biomass to degradation, and their bioconversion requires metabolic pathways that are distinct from those used to metabolize cellulose. In this review, we discuss the key differences in the structural features of xylans across diverse plant species, how these features affect their interactions with cellulose and lignin, and recent developments in understanding their biosynthesis. In particular, we focus on how the combined structural and biosynthetic knowledge can be used as a basis for biomass engineering aimed at developing crops that are better suited as feedstocks for the bioconversion industry.

## Background

Plant cell walls encompass the majority of terrestrial biomass and play many important environmental and economic roles [1]. Cell walls are complex structures that consist of cellulose, hemicellulose (xylans, xyloglucans, mannans, etc.), pectins, lignin, and some proteins [2, 3]. The amounts of each wall component can vary greatly depending on species, tissue, and cell type [2]. Xylans are the main hemicellulosic constituent found within the thickly lignified secondary cell walls of woody dicots such as poplar, and the primary and secondary cell walls of many monocot species, such as switchgrass, that are relevant to bioindustry [4]. Xylans in these tissues can account for up to 30% of the plant cell wall’s dry weight [5]. Melillo et al. have suggested that approximately 50 billion tons of carbon is incorporated by terrestrial plants annually [6]. If we modestly assume that across all species xylans account for approximately 20% of plant cell walls, then we conservatively estimate that roughly 10 billion tons of carbon is incorporated into xylan polymers annually.

In the biotechnology sector, particularly for the production of biofuels, xylans can present many challenges to efficient fermentation to useful products by contributing to biomass recalcitrance, defined as the resistance of biomass to chemical, thermal or enzymatic degradation. For one, xylans are composed mainly of pentose sugars, bioconversion of which requires metabolic pathways that are distinct from those used to process hexose sugars from cellulose [7]. Such systems for pentose utilization are often lacking in industrially relevant fermentative microbial strains [7]. Furthermore, the complexity of linkages and sidechain structures in xylan necessitate a suite of hydrolytic enzymes for the complete breakdown of the polymer, and the production of such enzymes can result in significant economic and metabolic costs. Finally, xylan is known to be highly substituted with *O*-acetyl groups, whose release leads to a reduction in pH that can have an inhibitory effect on fermentative microorganisms [8]. Thus, modification of xylans or specific xylan structures are of interest to the biomass-processing industry, as success in this area may facilitate fermentation and thereby substantially lower costs for full biomass degradation.

## Xylan structure

Xylans are defined as carbohydrate polymers consisting of a β-1,4-xylosyl (Xyl*p*) backbone, although xylans containing a β-1,3 and mixed linkage β-1,4-1,3 backbone structure have been found in algal species [9]. Many xylan’s structural characteristics, including its molecular mass and the identity and distribution of its substituents, vary considerably between species, cell type, and developmental stage. Nevertheless, xylans can be grouped into four major types: *O*-acetylglucuronoxylan (AcGX), arabinoglucuronoxylan (AGX), *O*-acetylglucuronoarabinoxylan (AcGAX), and *O*-acetylarabinoxylan (AcAX) [10].

**Fig. 1:**
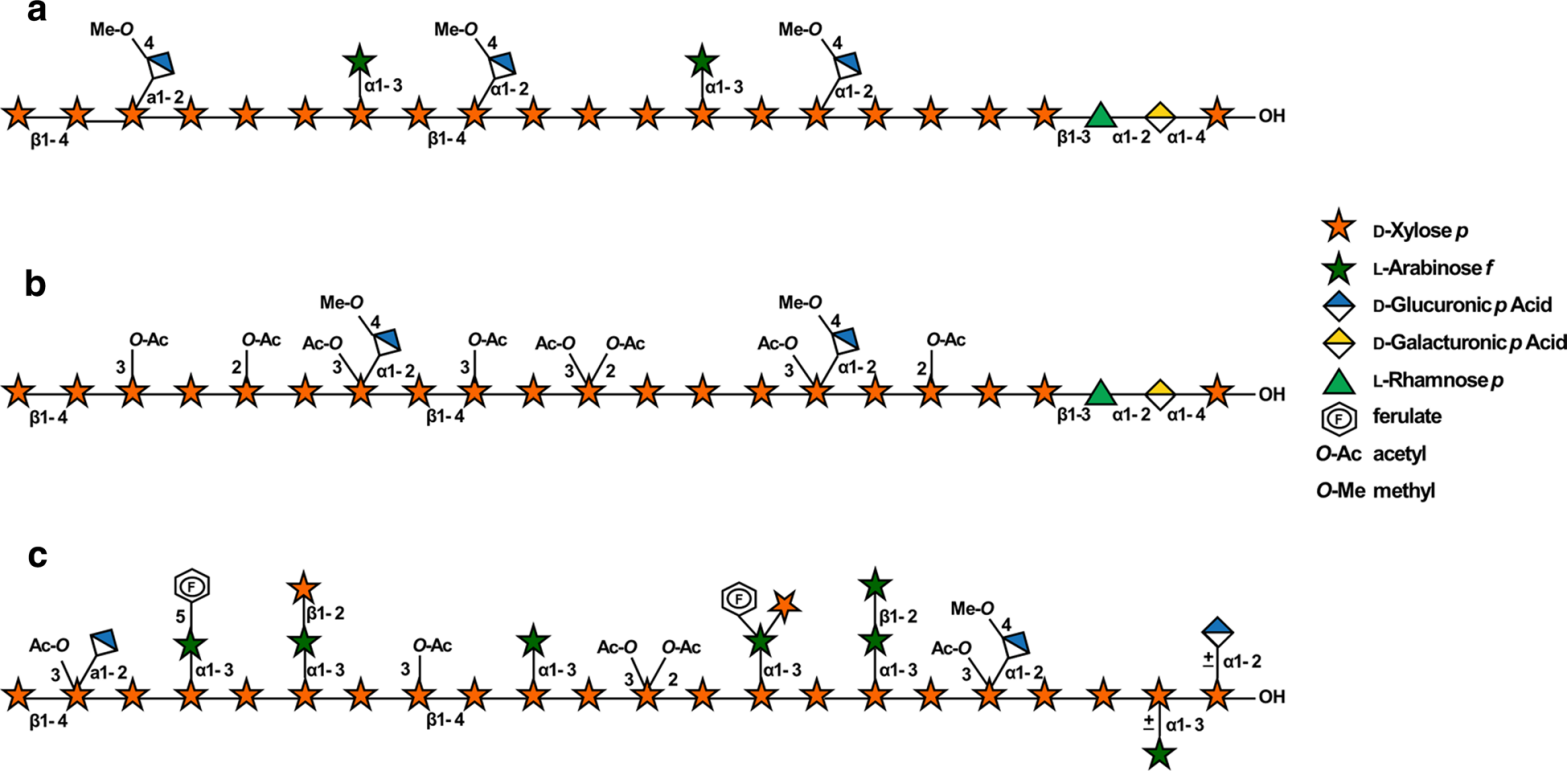
Xylan structures from spruce, poplar, and switchgrass secondary walls. Graphical representation of the main structural features of (**a**) arabinoglucuronoxylan (AGX) from spruce (**b**) acetylated glucuronoxylan (AcGX) from poplar, and (**c**) acetylated glucuronoarabinoxylan (AcGAX) from switchgrass. Spruce GX and poplar AcGX contain a distinct glycosidic sequence at their reducing ends, which is absent in switchgrass AcGAX, which often has substituted reducing xylosyl residues at the reducing end [25, 28, 43]. The GlcA and Ara substituents are in even positions and regularly distributed in the main domain of spruce AGX [27, 46]. The substituents in the main domain of Arabidopsis AcGX and poplar are also likely to be evenly distributed [22, 45]. The pattern of distribution of AcGAX substituents in switchgrass secondary walls is still unknown, but they are less branched than the AcGAX in primary walls and other tissue-specific grass xylans (see text for more details)

AcGXs are the predominant type of xylan found within the thick lignified secondary cell walls of hardwoods and herbaceous dicot species such as *Poplar* and the model plant *Arabidopsis thaliana* (Fig. 1) [11–13]. These AcGXs are homodisperse in length (approximately 100 residues in *Arabidopsis*) and, on average, one of every ten xylosyl residues is substituted at *O*-2 with (4-*O*-methyl)-α-d-glucuronic acid((Me)Glc*p*A) [13, 14]. In addition to glycosyl substitutions, the xylosyl residues in the backbone often bear *O*-acetyl esters, which are the most abundant substituents in AcGXs. For example, more than half of the backbone xylosyl residues in *Arabidopsis* and *Populus* AcGXs are *O*-acetylated [15–18]. These xylosyl residues can be mono-acetylated at *O*-2 or *O*-3 or di-acetylated at both *O*-2 and *O*-3, while the xylosyl residues carrying (Me)Glc*p*A at *O*-2 can also be acetylated at *O*-3. In *Arabidopsis* and *Populus* AcGXs, monoacetylated residues at *O*-2 or *O*-3 are the most abundant and account for 34 to 49% of all xylosyl residues. Only a small percentage of diacetylated residues are present (6–7%). Virtually all the xylosyl residues substituted with (Me)Glc*p*A at *O*-2 are acetylated at *O*-3 and these xylosyl residues account for approximately 10% of the total backbone residues [11, 15–20]. The ratio of 2-*O*- and 3-*O*-acetyl substituents in the xylan is difficult to determine since acetyl groups can migrate between the *O*-2 and *O*-3 positions of the same xylosyl ring [21]. This phenomenon has made it very challenging to determine the positions of these acetyl substituents when xylan is in the wall or while it is being synthesized in the Golgi. Recent studies of the *O*-acetylation distribution pattern in *Arabidopsis* indicated that every other xylosyl residue carries an acetyl ester, suggesting a systematic addition of *O*-acetyl groups to the GX backbone [16, 22].

Aside from backbone decorations, AcGXs contain a distinct tetrasaccharide sequence of Xyl*p*-1,4-β-d-Xyl*p*-1,3-α-l-Rha*p*-1,2-α-d-Gal*p*A-1,4-d-Xyl (termed Sequence 1) at the reducing terminus, though the biological function of this reducing sequence in the cell wall is still not known [14, 23]. Using this distinct sequence as a reference enabled us to determine that each GX polymer present in Arabidopsis and some hardwood species contain approximately 100 xylosyl residues [13, 14, 24].

Sequence 1 is also present at the reducing ends of coniferous arabinoglucuronoxylans [25]. These AGXs are substituted, on average, with two 4-*O*-methyl-α-d-glucuronic acid groups at *O*-2 and one α-l-arabinofuranose (Ara*f*) residue at *O*-3 per every ten xylose units, and are minor components of softwood cell walls [26]. These highly decorated AGXs found in the cell walls of most gymnosperms are generally not *O*-acetylated (Fig. 1). The exceptions are members of Gnetophyta, which synthesize *O*-acetylated xylans. These xylans also have other structural features typical of dicot AcGXs, such as undetectable levels of arabinosyl sidechains and low amounts of uronic acid substituents [27].

Xylans from monocot species show considerable structural diversity [28]. Grasses, which include grain (corn and rice) and energy crops (switchgrass and *Miscanthus*), are the most extensively studied of the monocots. The secondary cell walls of grasses contain AcGAX, which have Glc*p*A or MeGlc*p*A substituents at *O*-2; however, the main substitutions are α- l -Ara*f* residues at *O*-3. The α-l-Ara*f* residues are frequently further substituted at *O*-2 with α-l-Ara*f* or β-d-Xyl*p* residues (Fig. 1) [29, 30]. The backbone residues of AcGAXs in primary walls are singularly or doubly substituted with α-1-2 and/or α-1-3 linked arabinosyl residues [31]. High molecular mass neutral AcAX, without uronic acid substituents, can be found in the cell walls of starchy cereal grains [10]. Some grasses contain more complex xylans in specific tissues, for example, AcGAXs in corn bran and corn fiber contain complex sidechains with sugars that are not typically found in xylans, such as α-l-galactose and α-d-galactose [32].

**Fig. 2:**
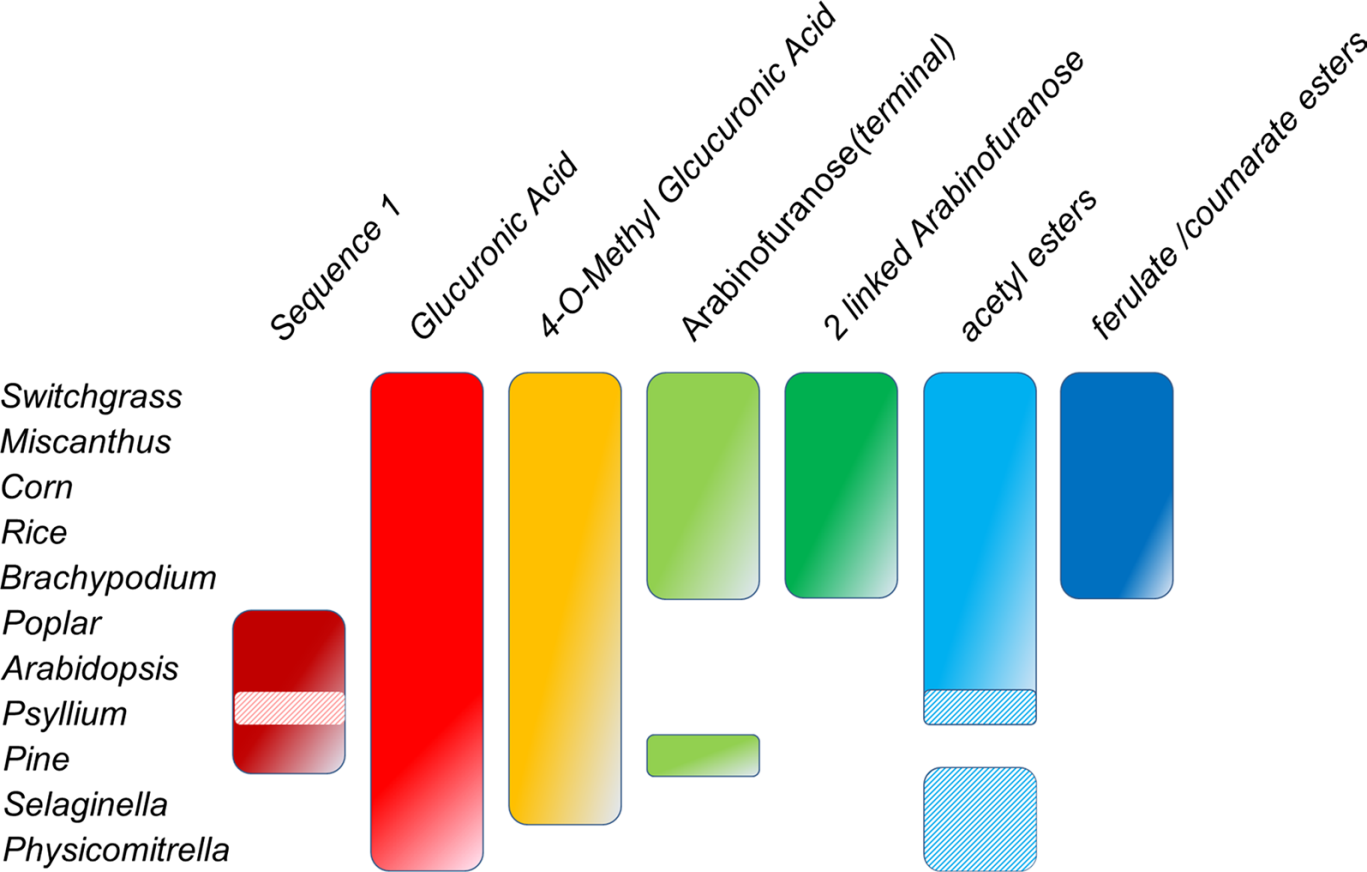
Structural features of xylans in bioindustry crops and model organisms. Structural features of xylans from model and industrially relevant plant species. Bars represent detectable amounts of these features described in the literature. Dashed bars represent a lack of analysis describing the presence or absence of these structures. Other structural features not shown may also be present on xylans isolated from these species

Grass AcGAXs and AcAX are acetylated but to a lesser extent than AcGXs from dicots. However, in addition to the acetyl groups attached to the backbone xylosyl residues, the Ara*f* substituents can also carry acetyls at *O*-2 [33]. A notable feature of grass AcGAX and AcAX is that their Ara*f* residues are often esterified with ferulic or *p*-coumaric acids at *O*-5 [34, 35]. Oxidative coupling of ferulic acid substituents leads to the formation of ferulate dimers or trimers, which crosslink different xylan molecules or xylan to lignin [36, 37]. Further, it has been proposed that the ferulates are the initiation sites for cell wall lignification in grasses, making them another interesting target for biomass modification [38, 39] (Fig. 2).

The reducing-end tetrasaccharide, Sequence 1, which is characteristic of xylans from dicots and gymnosperms, has not been detected in xylans isolated from grasses (Fig. 2). Instead several different structures were found at the reducing terminus of grass AcGAX and AcAX, including specifically substituted xylosyl residues at the reducing end of the polymer [28, 40]. However, the presence of Sequence 1 in xylans synthesized by some commelinid monocots and its absence in xylans from some non-commelinid species indicate that the structural diversity of xylan in the monocots is greater than what was previously thought [31]. Interestingly, some non-commelinid species (*Asparagales* and *Alismatales*) synthesize xylans that lack the reducing-end tetrasaccharide sequence and are substituted with the disaccharide sidechain Ara*p*-1,2-α-(Me)GlcA [28]. This sidechain is also found in xylans isolated from *Eucalyptus* wood and *Arabidopsis* primary cell walls, suggesting a potentially conserved structural or biosynthetic role of primary cell wall xylans within evolutionarily distant species [28, 41]. Xylan present in woody tissues of *Eucalyptus* contains sidechains comprised of β-d-Gal*p* attached at *O*-2 of the MeGlcA residues, in addition to the α-l-Ara*p*-containing disaccharides [17]. Xylan that is highly substituted with more complex sidechains can be found in some seed mucilage and root exudates [10]. For example, the xylan in the mucilage of *Arabidopsis* seeds contains sidechain xylosyl residues attached directly to the backbone [42].

Xylans are essential components of the thick and strong secondary walls of the specialized cells that constitute fibers and conducting vessels in vascular plants. However, the presence of xylans in the cell wall precedes plant vascularization, and xylan that is structurally similar to secondary wall GX has been found in small amounts in the avascular moss *Physcomitrella* [43]. In contrast to the GXs from Poplar and other woody species, in which a majority of the GlcA substituents are methyl etherified at *O*-4 [11], the xylan in *Physcomitrella* is not methylated [43], suggesting that *O*-methylation of GXs is a key structural feature of the secondary cell walls of vascular plants. In herbaceous dicots, the extent of 4-*O*-methylation of the GlcA residues varies depending on the tissue type and growth conditions. Interestingly, differential binding of a MeGlcA-specific carbohydrate binging module (CBM) has demonstrated that GX in the vascular xylem of *Arabidopsis* has a higher degree of methylation than in interfascicular fibers, which further supports the relationship between high GX methylation and highly lignified hydrophobic walls [44].

Another structural characteristic that affects xylan properties is the spacing between GlcA, *O*-acetyls or other substitutions, which is believed to be a strictly controlled feature of xylans in dicot and conifer species [16, 45]. Recent studies have suggested that xylans may contain domains with distinct GlcA spacing, and that these variations may result in different xylan conformations in vivo [27, 45]. This has led to the two domains on *Arabidopsis* xylan being termed the major domain, where GlcA residues are spaced at approximately 10 backbone xylosyl residues from one another at even intervals, and the minor domain where these substituents are much closer (5–7 residues), and have no preference for even or odd spacing [45]. Similar domains have been proposed for conifer xylans [27]. In spruce xylan, a main domain containing evenly spaced GlcA substitutions and frequent *Ara* substituents that are approximately two residues apart was identified, along with two other minor domains [46]. However, the question still remains whether these domains are part of the same xylan molecule or represent different xylans with distinct structural features [46].

## Xylan interactions with cellulose and lignin

Xylans are structurally similar to cellulose in that their backbones are composed of 1-4-linked xylosyl residues that have equatorial oxygen atoms at both C1 and C4. This common sugar geometry results in polysaccharide backbones with molecular shapes that are complementary to cellulose [23]. As indicated previously, xylans spontaneously bind to cellulose microfibrils produced by *Acetobacter xylinum*, providing evidence that the physical property of xylans can affect cellulose orientation and aggregation during cell wall assembly [47]. For example, in situ labeling experiments of woody tissues have demonstrated a preferential localization of AcGX in the transition zones between the S layers, where the cellulose changes orientation, supporting the hypothesis that AcGX participates in organizing cellulose microfibrils into a helicoidal arrangement [48–50].

Certainly, the type and distribution of the backbone substitutions have important effects on the binding interactions of xylan with itself and other polymers in the wall. It has been reported that sparsely branched xylans have a higher affinity for cellulose microfibrils, and that even small *O*-acetyl substituents have pronounced impacts on the adsorption of xylans to cellulose [51–53]. In contrast, recent studies using molecular dynamics simulation indicate that xylan substitutions stabilize rather than limit the binding of xylan to cellulose. These seemingly contradictory results were rationalized by proposing that the increased absorption of sparsely substituted xylans occurs because a low degree of substitution leads to the self-association of xylans, causing additional xylan molecules to aggregate with xylan molecules that are directly bound to cellulose [46, 54].

Current models predict that the threefold helical screw conformation that xylan adopts in solution shifts to a flat helix with twofold screw symmetry when xylan interacts with cellulose [55]. It was proposed that GlcA and/or *O*-acetyl substituents that are separated by an even number of backbone residues, and thus decorate only one side of the xylan ribbon, facilitate the formation of hydrogen-bond networks between xylan and hydrophilic cellulose surfaces. A model was proposed in which the substituents of such xylans point away from the cellulose fibrils, while attachment of substituents to both sides of the ribbon would hinder the interactions between xylans and the hydrophilic surfaces of cellulose [22, 55]. In the case of the hydrophobic surface, however, one model suggests that consecutive substitutions strengthen the binding of xylan with cellulose [46].

In addition to interacting with cellulose, xylans are physically and/or covalently bound to lignin in secondary cell walls of lignocellulosic biomass to form a closely associated network [38]. Strong evidence indicated that GAXs in the secondary walls of grasses are crosslinked into lignin by extensive copolymerization of their ferulates [56–58]. In the case of hardwoods and other dicots, it has been proposed that GXs are esterified to lignin via their MeGlc*p*A substituents [59, 60]. However, only indirect evidence has been reported to support this hypothesis. Lignin-carbohydrate complexes have been isolated from numerous woody species, but much remains to be learned about the molecular structure of these complexes [61]. Further, recent studies on *Populus* genotypes with different cell wall compositions suggest that there is a close interaction between lignin and xylan, and that the degree of xylan acetylation influences the interaction between these major cell wall polymers, affecting the efficiency of pretreatment with 0.3% H_2_SO_4_ in nonisothermal batch reactors [62].

## Enzymes involved in xylan synthesis

Through the diligent work of many different research groups over many years, several of the glycosyltransferases (GT’s) responsible for xylan synthesis have been brought to light. Initial research in this field focused on the observed biochemical and phenotypic effects of xylan biosynthetic mutants in the model dicot species *Arabidopsis thaliana*. Many of these so-called *irregular xylem* (*irx*) mutants displayed a collapsed or irregular xylem phenotype resulting in stunted growth and often infertility [63]. Structural analysis of GX isolated from *irx* mutants, combined with biochemical analysis of the associated gene products, has led to the characterization of enzymes involved in many aspects of xylan synthesis in dicots including backbone elongation [64–66, 72], sidechain addition [45, 67–69], reducing-end synthesis [14], and noncarbohydrate modifications such as the addition of acetyl [20, 64, 70], and methyl groups [44].

**Fig. 3:**
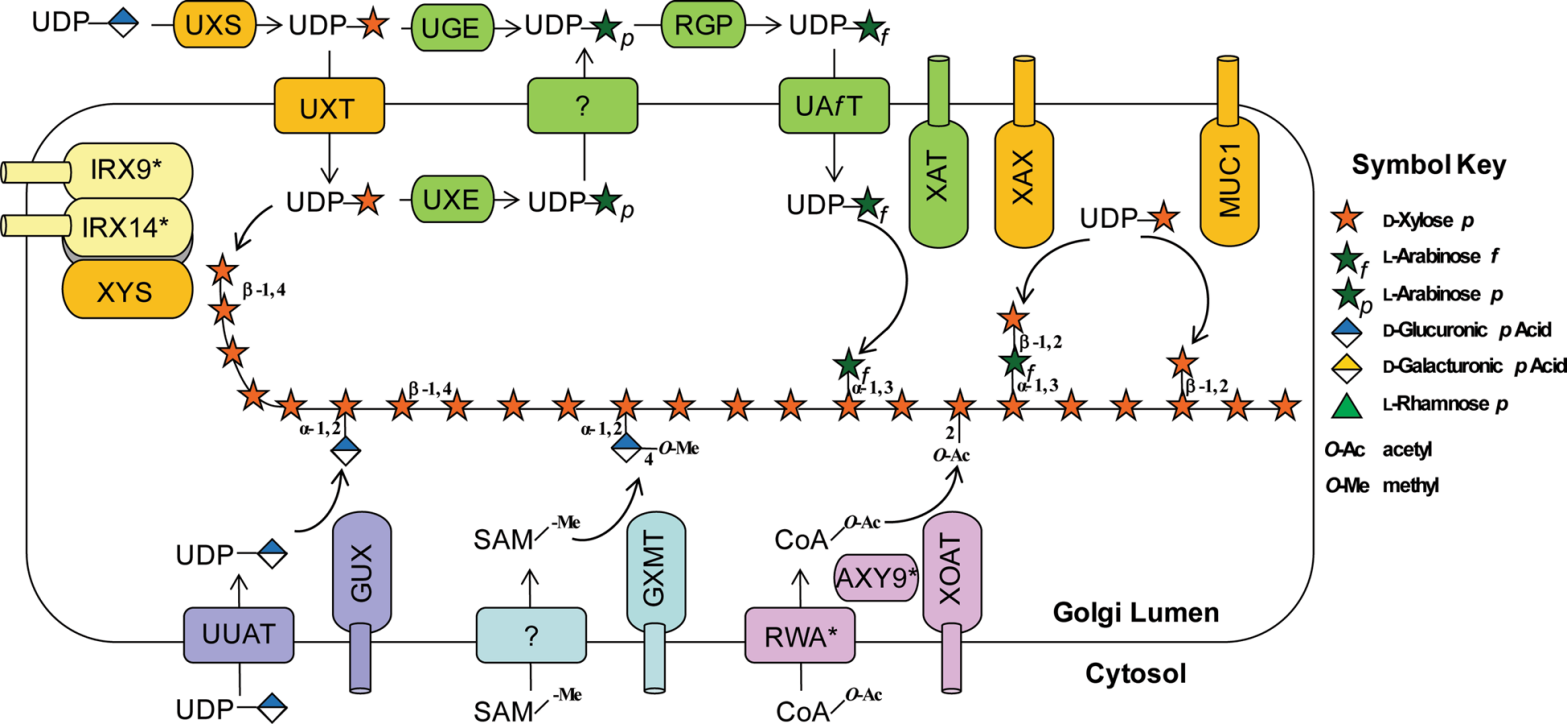
Schematic model of xylan biosynthesis. Xylan biosynthesis takes place in the Golgi lumen. This process requires the generation and transport of several activated nucleotide sugars in addition to both *O*-acetyl and methyl donors. UDP-Xyl is generated via decarboxylation of UDP-glucuronic acid by UDP-xylose synthase (UXS) in the cytosol, and then transported into the Golgi lumen by UDP-Xyl transporters (UXT) [115]. Synthesis of the xylan backbone is catalyzed by XYS, which is part of a Golgi-localized xylan synthase complex (XSC) that also includes IRX9 and IRX14; however, the roles of the latter enzymes in this process remains enigmatic. UDP-GlcA is transported into the Golgi by a UDP-uronic acid transporter (UUAT) protein [116], and then GUX enzymes catalyze the transfer of GlcA from UDP-GlcA to the xylan backbone, which is subsequently methyl-etherified by GXMT proteins. For the addition of Araf residues, C-4 epimerization of UDP-Xyl to UDP-Arap is carried out by a Golgi-localized UDP-Xyl 4-epimerase (UXE) or cytosolic UDP-glucose 4-epimerases (UGE) [117]. UDP-Arap produced in the Golgi is either used as a substrate in the synthesis of Ara*p* containing polysaccharides such as pectins, or transported back to the cytosol via an unknown process. In the cytosol, UDP-Ara*p* is interconverted to UDP-Ara*f* by UDP-Ara mutases (reversibly glycosylated polypeptide, RGP) [118], and is then transported back into the lumen of the Golgi apparatus by UDP-Ara*f* transporters (UAfT) [119]. XAT enzymes then catalyze the addition of Ara*f* residues to *O*-3 of the xylan backbone, which is often further substituted by a β-xylosyl residue to *O*-2 by XAX enzymes. The xylan present in Arabidopsis seed mucilage is also decorated with β-xylosyl residues at *O*-2, which are added by the xylosyltransferase MUC1. Acetyl donors, such as Acetyl-CoA or an unidentified acetyl donor, are most likely imported into the Golgi lumen by RWA proteins, and then acetylation of the xylan backbone occurs via a number of xylan acetyltransferases (XOAT), which have different catalytic regiospeficities. * Indicates that activity has not been biochemically confirmed

In contrast to the well-known cellulose synthases, which are localized to the plasma membrane of plant and bacterial cells, most enzymes responsible for xylan synthesis are found as membrane-associated proteins within secretory organelles [i.e., endoplasmic reticulum (ER) and the Golgi apparatus] [71]. Hemicellulosic polymers, including xylan and xyloglucan, are synthesized primarily in the Golgi and then exported via poorly characterized mechanisms to developing cell walls. Many of the enzymes involved in xylan synthesis are from distinct carbohydrate-active enzyme (CAZy) GT families [72]; however, they are thought to interact and form dynamic protein complexes within the Golgi and function in a concerted manner to form complex hemicellulosic structures [71]. A proposed model of xylan synthesis is presented in Fig. 3.

## Enzymes involved in backbone elongation

Three proteins (and their homologs) have been implicated in xylan backbone synthesis in dicot and monocot species, including IRX9 and IRX14, in the GT43 family, and IRX10/IRX10-L, in the GT47 family. IRX10/IRX10-L proteins have recently been shown by two groups to possess β-1,4-xylosyl transferase activity in vitro when expressed heterologously in either human embryonic kidney293 (HEK293) cells or *Pichia pastoris* [64, 73]. Using HEK293-based expression, *At*IRX10-L, now renamed to xylan synthase 1 (XYS1), was able, via a distributive mechanism, to transfer xylosyl residues from UDP-xylose to labeled xylo-oligosaccharides as small as xylobiose, and to extend a xylohexaose primer to form products up to 21 xylosyl residues in length [64]. This result came as somewhat of a surprise given that the backbones of all other hemicelluloses with geometric homology to cellulose are synthesized by enzymes belonging to family GT2, which contains the cellulose synthase superfamily. Family GT2 glycosyltransferases are multi-membrane spanning proteins that polymerize polysaccharides processively with simultaneous excretion through the membrane [74]. This is in stark contrast to the GT47 *At*XYS1, which does not appear to even contain a transmembrane domain [75], and acts via a distributive mechanism in vitro [64].

IRX9 and IRX14 are also believed to play a role in xylan backbone elongation based on work with mutants that indicated that they are essential for formation of the complete backbone *in planta* [14, 71, 76]. Further experiments with microsomal membrane preparations have shown that xylosyl transferase capacity is reduced in microsomes prepared from mutants (*irx9* or *irx14*) of either of these two proteins [71]. However, in vitro analysis using techniques that were employed to demonstrate xylosyltransferase activity of XYS1 have failed to show any xylan synthase activity for these enzymes, whether alone or in combination [64]. Both enzymes are classified as members of the GT43 family; however, it remains unclear if these proteins are themselves catalytic, or if they simply serve as structural components of a larger xylan synthase complex (XSC) or function as accessory proteins that facilitate the transfer from XYS1 to the growing xylan chain. For example, in *At*IRX9 the catalytically important DxD motif present in most GTs in the GT-A fold family is replaced by an unusual amino acid sequence (‘GLN’). Moreover, the closely related protein IRX9-L has ‘DDD’ in this position [76]. Interestingly, Ren et al. used site-directed mutagenesis and genetic complementation to show that *irx9* null mutants could be successfully complemented by a modified IRX9-L gene in which the ‘DDD’ motif was changed to ‘ADA’ [76]. Further, recent work with heterologously expressed *Asparagus officionalis Ao*IRX10, *Ao*IRX9, and *Ao*IRX14 in *Nicotiana benthaliama* demonstrated that these three proteins form a Golgi-localized XSC in vivo [66]. However, the exact role of each protein in the complex is still not well understood. Mutagenesis experiments affecting the DXD motif of each putative GT, which should disable the protein’s catalytic capacity, showed that this motif was essential for *Ao*IRX10 and *Ao*IRX14 activity. However, no decrease in xylosyl transferase activity was observed upon analysis of microsomes containing *Ao*IRX9 in which critical catalytic residues had been replaced [66]. Bimolecular fluorescence complementation (BiFC) analysis with the *Asparagus* proteins also provided the first direct evidence that *Ao*IRX9, *Ao*IRX10, and *Ao*IRX14A are members of a core XSC localized in the Golgi that likely contains additional proteins [66]. Taken together, these data suggest that IRX9 does not have a direct catalytic role in xylan synthesis, but rather plays a structural or supportive role in the XSC. However, no functional in vitro characterization of any of the GT43 enzymes involved in plant polysaccharide synthesis has yet been reported, therefore their exact role in the XSC remains enigmatic.

## Enzymes involved in synthesis of the reducing-end structure (Sequence 1)

As mentioned previously, xylans from dicots and some monocot species often contain a distinct tetrasaccharide motif termed Sequence 1 at their reducing ends [14, 28]. The role of this structure in xylan synthesis is still poorly understood, and the biosynthetic mechanism for its creation has remained elusive. Mutagenic experiments in *Arabidopsis* have presented some candidates for Sequence 1 biosynthesis as this structure is lacking in xylans from plants deficient in certain secondary cell wall expressed proteins. Thus, IRX7/FRA8 (GT47), IRX8/GAUT12 (GT8), and PARVUS/GATL1 (GT8) are the main glycosyltransferase candidates for synthesis of this unusual structure, although concrete biochemical evidence to support their participation in this process is still lacking [3].

**Fig. 4:**
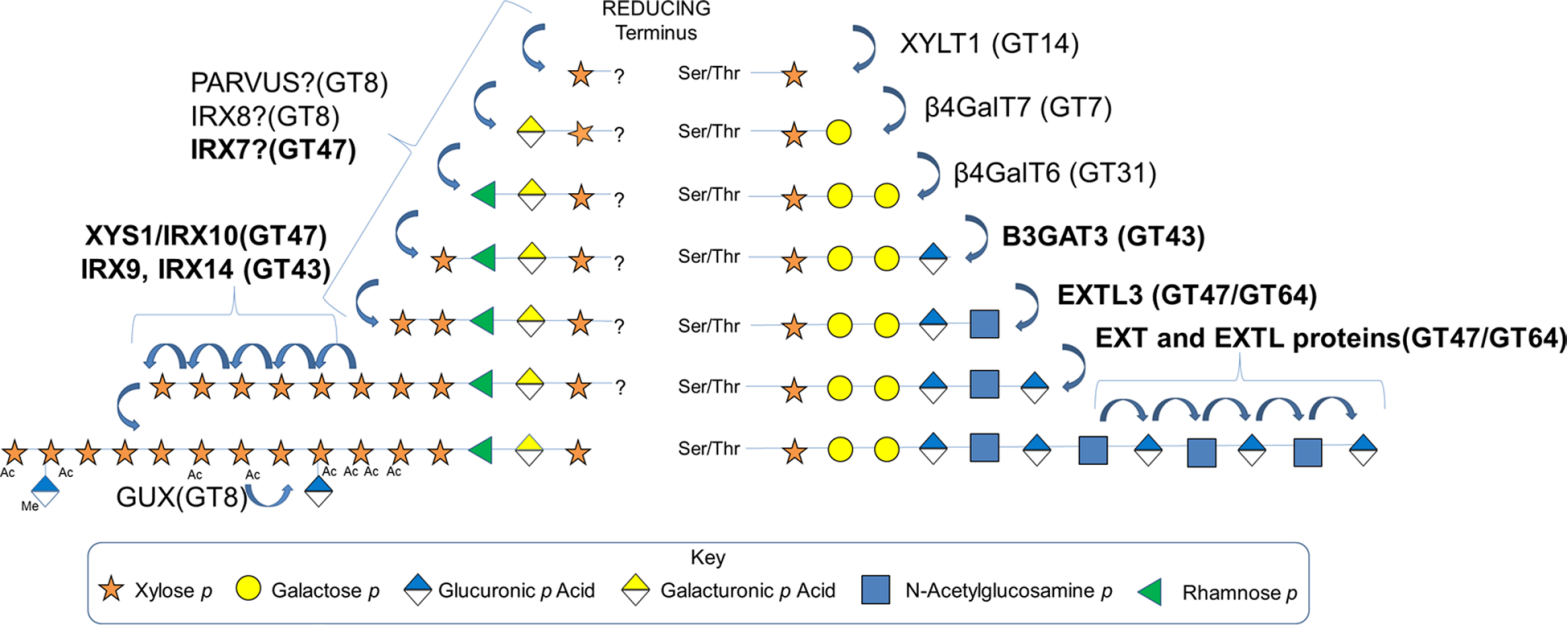
Models of glucuronoxylan and heparan sulfate biosynthesis. Comparison of proposed models of xylan and heparan sulfate biosynthesis. In bold are enzymes from the families’ common between the two pathways (GT43 and GT47). In heparan sulfate biosynthesis, polysaccharide initiation occurs by the transfer of a xylosyl residue to a protein serine or threonine residue by the enzyme xylosyl transferase 1 (XYLT1) [77]. A linker tetrasaccharide is then synthesized by the enzymes β-1-4 galactosyl transferase 7(β4GalT7), β-1-4 galactosyl transferase 6(β4GalT6) and a GT43 family enzyme Galactosylgalactosylxylosylprotein 3-β-glucuronosyltransferase 3(β3GAT3). Following primer synthesis, the polymer is extended by the GT47/64 heparan synthases, exotosin (EXT) and exotosin-like (EXTL3) proteins, which catalyze the transfer of the repeating segment of glucuronic acid (GlcA*p*) and *N*-acetyl glucosamine (GlcNAc*p*) [77]. This mechanism has similarities to our proposed model for xylan synthesis, where a tetrasaccharide primer may be synthesized while connected to some unknown carrier in the ER/Golgi, potentially in part by GT47 and GT43 family enzymes. This primer is then extended by the GT47 XYS1/IRX10 family of proteins, which most likely function as part of protein complexes that also contain members of GT43 (IRX9, IRX14). The xylan chains are then decorated with sidechains such as acetyl esters and glycosyl units such as (Me) GlcA*p*

The role of Sequence 1 in xylan synthesis also remains an enigma. Many have speculated that Sequence 1 may be serving as a terminator of xylan synthesis, given the observation that deregulation of xylan chain length occurs when Sequence 1 synthesis is disrupted [14, 23]. However, the recent characterization of the xylan backbone synthase (XYS1) has shown that xylosyl addition occurs from the reducing end to the nonreducing end, making the case for a reducing-end terminator unlikely [64]. Further, it is interesting to note that many of the enzyme families involved in xylan synthesis, such as GT47 and GT43, also function together in the biosynthesis of animal glycosaminoglycans (GAG), such as heparan sulfate and chondroitin sulfate, which are charged and heavily sulfated polysaccharides that play many vital roles in animal biology. These polysaccharides require the synthesis of a tetrasaccharide primer before elongation of the GAG backbone can occur. In the case of GAG synthesis, however, the polysaccharide is known to be covalently linked to a serine or threonine of a protein-based acceptor [77]. It is unclear if xylans are linked at the reducing terminus to a protein or lipid in the Golgi apparatus and released at a later time. A proposed model of xylan synthesis is contrasted with that of the biosynthesis of the GAG heparan sulfate in Fig. 4.

## Proteins involved in the addition of glycosyl substituents

The roles of several enzymes in the addition of sidechains to the xylosyl backbone have been elucidated in recent years. Three members of GT family 8, GlucUronic acid substitution of Xylan 1 (GUX1), GUX2, and GUX3, have been shown to possess glucuronosyltransferase activity toward xylooligimers, and *Arabidopsis* mutants lacking these enzymes result in xylans with reduced GlcA and 4-*O*-MeGlcA substitutions [41, 45, 68, 69]. Further evidence suggests that GUX1 and GUX2 perform distinct functions in decorating the xylan backbone regions, leading to different spacing between GlcA residues. GUX1 is proposed to be responsible for forming the xylan major domain by adding GlcA substitutions about every 10 xylosyl residues, whereas GUX2 has been proposed to decorate segments comprising the minor domain by placing the GlcA residues closer together (6–8 residues) [45]. GUX3 has also been shown to play a defined role by acting as the sole transferase required for GlcA sidechain addition to xylans that are incorporated into the primary cell walls of Arabidopsis [41].

Enzymes involved in the decoration of the arabinoxylan backbone with arabinosyl and xylosyl sidechains have been shown to be members of the GT61 family, which is divided into three clades: A, B, and C [78]. The Xylan Arabinosyl Transferases (XATs) responsible for addition of Ara*f* to *O*-3 of the xylan backbone have been identified in grasses and are members of GT61 clade A. Heterologous expression of XAT in *Arabidopsis* resulted in the arabinosylation of *Arabidopsis* GX, which normally does not possess Ara*f* residues [78]. It is unclear how many enzymes are required to complete the full suite of arabinosyl substitutions found on monocot xylans, given that residues can be arabinosylated at O2, O3, or in both positions. Xylosyl Arabinosyl substitution of Xylan 1 (XAX1), another GT61 enzyme in the grass-specific clade C.IV, has been implicated in the addition of β-xylosyl residues to O2 of α-1,3-Ara*f* residues decorating the xylan backbone [67]. It was also suggested that transfer of xylose enhances feruloylation of the α-1,3-Ara*f* residues, or that feruloylation interferes with hydrolysis of this xylosyl residue during xylan maturation [67]. A forward genetics screen applied to a mutant population of *Brachypodium distachyon* identified a SNP in Bradi2g01480 (SAC1), a member of grass-specific clade C.III of the GT61 family, that impacts biomass digestibility. Xylan enriched fractions isolated from *sac1* plants have less xylose, indicating that SAC1 may have a similar function to XAX1 from rice [79]. Recently, a mutant in MUCILAGE-RELATED 21 (MUCI21), a putative xylosyl transferase in clade B of the GT61 family, was shown to be involved in the synthesis of seed mucilage xylan. Analysis of mucilage from *muci21* plants suggests that this enzyme catalyzes the transfer of a β-1,2 xylosyl residue directly to the xylan backbone [42].

## Proteins involved in non-glyosidic decorations

### 4-*O*-methylation

As discussed previously, a variety of non-glycosyl substitutions are also present in xylan. One of the best characterized of these is the 4-*O*-methylation of GlcA sidechains. The enzymes responsible for this modification in *Arabidopsis* were initially identified as Gluruconoxylan Methyl Transferase (GXMT) proteins by researchers in the BioEnergy Science Center [44, 80]. Three homologs of these proteins have been studied in *Arabidopsis,* all containing a Domain of Unknown Function 579 (DUF579). Recombinantly expressed GXMT1 was able to catalyze the transfer of a methyl group from *S*-adenosyl methionine to the 4 position of GlcA residues present on GX polymers and oligosaccharides [44]. Interestingly, the disruption of normal xylan synthesis in mutants of many of the GT enzymes mentioned previously often leads to an increase in the ratio of methylated to un-methylated GlcA residues in GX [14]. One possible explanation for this is that when xylan synthesis is reduced, pools of methyl donor accumulate, while the concentration of glucuronosyl acceptors is reduced, leading to an increase in the extent of their methylation. Another theory is that slowing of xylan synthesis in biosynthetic mutants provides more time for the methyl transferases to interact with their acceptor substrates. Further characterization of this phenomenon should provide insight into the overall process of xylan biosynthesis.

### Ferulic acid and *p*-coumaric acid esters

Some of the arabinofuranosyl residues of monocot xylans are also decorated at *O*-5 with ferulic or *p*-coumaric acid esters. Ferulic substituents form oxidatively-linked dimers and oligomers with wall polymers that result in a covalently linked network within the wall. Although the process by which these modifications are added to the polysaccharide is still poorly understood, recent work has suggested that members of the “Mitchell clade” within the BAHD acyltransferase superfamily are involved in ferulic and p-coumaric acid esterification of monocot xylans [81–83]. These enzymes have been shown to localize to the cytoplasm, suggesting that other players are important in this process to complete ferulic acid transfer, which most likely takes place in the Golgi. It’s likely that feruloyl-CoA is the primary feruloyl donor in vivo; however, it remains unknown whether the feruloyl moiety is transferred directly to arabinoxylans or to another intermediate, such as UDP-Ara*f*. It has been hypothesized that ferulic acid is first transferred to a glycosyl donor such as UDP-Ara*f* in the cytoplasm, and then feruloylated UDP-Ara*f* is transported into the Golgi where transfer of feruloylated Ara*f* onto the xylan backbone may occur [3].

Recently, Marcia and coauthors showed that downregulation or overexpression of *Bd*AT1, a member of the “Mitchell clade” of BAHD acyltransferases in *Brachypodium* resulted in reduced or increased levels of monomeric and dimeric ferulic acid esters, respectively [84]. Taken together, their data indicates that *Bd*AT1 is a promising candidate for feruloylation of AX in grasses. Many intermediate steps in this process are still unknown, but when elucidated, will provide several interesting targets for biomass modification.

### *O*-Acetylation

*O*-Acetylation is one of the predominant modifications of xylan, and at least four protein families are involved in the cell wall polysaccharide acetylation pathway in the plant Golgi. These are Reduced Wall Acetylation (RWA) proteins [85], Trichome Birefringence-Like (TBL) proteins [86], the Altered XYloglucan 9 (AXY9) protein [87], and GDSL acetylesterases [88]. The RWA2 protein was the first protein shown to be involved in cell wall acetylation in plants and was identified in *Arabidopsis* based on its homology to the Cas1P protein, which is involved in polysaccharide *O*-acetylation in the pathogenic fungus *Cryptococcus neoformans* [85]. Mutation of the *RWA2* gene resulted in a 20% reduction of acetylation across several polysaccharides, including pectins, xyloglucan, and xylan [85]. RWA2 belongs to a family of four proteins in *Arabidopsis*. Using combinations of multiple *rwa* mutants, Manabe et al., demonstrated that RWA proteins have overlapping functions, and any one of the four proteins is able to support some level of acetylation of all polysaccharides in the wall [89]. Shortly after the identification of the RWA family, the plant-specific TBL family was shown to be involved in acetylation of specific cell wall polysaccharides [86]. Analysis of plants bearing mutations in the *TBL29* gene (also known as *ESKIMO1, ESK1*), which is highly expressed during secondary cell wall biosynthesis, has provided insights into its role in vivo. The xylan isolated from *tbl29*/*esk1* mutants has reduced amounts of mono-acetylated xylosyl residues, suggesting an essential role in xylan *O*-acetylation [20]. Moreover, in vitro biochemical analysis of the TBL29/ESK1 protein by researchers in the BioEnergy Science Center established the precise molecular function of these plant-specific proteins: i.e., the *O*-acetylation of xylan backbone residues [64]. In addition to TBL29/ESK1, the other eight members of the TBL family in *Arabidopsis* have been recently biochemically characterized and shown to possess xylan acetyltransferase activities in vitro. TBL28, TBL30, TBL3, TBL31, TBL34, and TBL35 are responsible for mono-acetylation at *O*-2 or *O*-3 and/or di-acetylation at both *O*-2 and *O*-3 of xylosyl residues, while TBL32 and TBL33 transfer acetyls at *O*-3 of xylosyl residues substituted at *O*-2 with (Me)GlcA [90].

TBL proteins are composed of one *N*-terminal transmembrane domain and two conserved domains, the TBL domain, and a domain of unknown function 231 (DUF231) [91]. The TBL domain harbors a conserved Gly-Asp-Ser (GDS) motif, and the DUF231 domain contains an Asp-x-x-His (DxxH) motif in the carboxy-terminus [92]. It has been hypothesized that one of the two domains binds the polymer while the other facilitates the binding of the acetyl-donor, and then transfers the acetyl group to the polysaccharide acceptors [92]. TBL proteins are predicted to be members of the GDSL-like family based on the presence of these conserved motifs [93]. Members of the GDSL esterases/lipases family harbor a”GDSL” sequence motif that is highly conserved across all kingdoms. GDSL hydrolytic enzymes are functionally diverse, and have been shown to act as proteases, thioesterases, arylesterases, and lysophospholipases [93]. GDSL esterases/lipases belong to the SGNH hydrolase superfamily, which is characterized by four conserved sequence blocks (I, II, III, and V) that were first used to describe the lipolytic enzymes [94]. The GDSL motif is part of block I, where the Ser residue is suggested to form a catalytic triad with the aspartate and histidine residues in DxxH motif in block V [95, 96]. Mutations of the GDSL and DxxH in *Arabidopsis* ESK1 were found to lead to a complete loss of xylan acetyltransferase function [90]. A rice GDSL protein, Brittle leaf Sheath 1 (BS1), has recently been reported to function as an acetyl xylan esterase, which is the first member of the GDSL family in plants that has polysaccharide esterase activity [88]. This conclusion is supported by the observations that recombinant BS1 functions as an esterase in vitro and backbone residues of xylan isolated from *bs1* mutants display increased acetylation at *O*-2 and *O*-3 [88].

Taken together, these data suggest that RWA proteins operate at a biosynthetic step preceding those of the AXY9 and TBL proteins, and because of their overlapping specificities they are predicted to function in the transport of acetyl donors into the Golgi (Fig. 3). AXY9 is hypothesized to function in an intermediate step between RWA proteins and the TBL acetyltransferases, and may act to shuttle unidentified acetyl donors. Finally, the ability of the BS1 enzyme to modulate xylan acetylation via its acetylxylan esterase activity in the Golgi suggests that it plays a role in maintaining acetylation levels and or patterning on the xylan backbone. RWAs, TBLs, and BS1 provide several potential targets for genetic engineering to improve biomass by altering xylan acetylation.

## Xylans as a target to reduce recalcitrance

Xylans are highly abundant polysaccharides in plant secondary cell walls and play a major role in the recalcitrance of crops grown as feedstocks for bioprocessing and bioenergy applications. However, developing strategies to modify xylans that minimize these recalcitrance barriers while simultaneously retaining plant fitness has been very challenging. This is due in part to the largely unpredictable pleiotropic effects of many xylan pathway mutations, combined with severe growth phenotypes associated with these mutations. For example, RNAi silencing of IRX8/GAUT12 in *Populus*, an enzyme implicated in the biosynthesis of GX Sequence 1, affects GX structure, GX abundance, and levels of pectic polysaccharides [97]. Interestingly, biomass from these plants was less recalcitrant and cell wall polymers were more easily extracted from its cell walls. However, it has been difficult to determine if the *primary* cause of these characteristics was a change in the structure or overall abundance of xylan or pectin [97]. Attempts to silence or knock out expression of other enzymes implicated in Sequence1 biosynthesis, including IRX7/FRA8 [12, 98] and PARVUS/GATL1 [99, 100] in *Arabidopsis* and *Populus*, resulted in plants with a reduced overall growth, rendering mutants such as these poor choices for use as industrial feedstocks. Given the reports regarding previous attempts to modify xylan structure for increased yields, suggesting that it will be more effective to engineer xylan in which the structures, abundances or spatial distributions of specific sidechains are modified (i.e., substituent engineering) to facilitate bioprocessing.

In biomass-accumulating secondary cell walls, gene expression is controlled by a signal transduction network involving various transcription factors, including secondary wall NAC-domain master switches and their downstream transcription factors [101–103]. The distinct expression patterns of different NAC genes in specific cell types potentiates their promoters as tools for spatial manipulation of polysaccharides in modified biomass for improving biofuel production. For example, the dwarf phenotype of Arabidopsis irregular xylem (*irx*) mutants was rescued by expressing the corresponding xylan synthesis-related genes in vessels using Vascular Related NAC Domain 6 (VND6) and VND7 promoters, which produced transgenic lines with lower xylan and lignin contents, and improved saccharification yields [104]. Thus, a promising strategy to modify cell walls for improved biomass is the use of cell type-specific overexpression or silencing of particular genes of interest. As the regulatory elements influencing the expression levels of certain gene products are characterized, and next generation genome editing techniques such as CRISPR-CAS9 are gradually being realized, manipulation of certain cell wall metabolic enzymes in the right place at the right time is finally becoming practical. Future efforts will utilize promoters that can be induced in specific cell types (e.g., fiber or vessel cells) to control the expression of genes known to impact xylan structure while avoiding undesirable growth phenotypes that often result from the use of constitutive promoters. Utilizing such precise strategies to control gene expression should lessen the detrimental effects of these mutations, thus increasing plant fitness.

Another approach that may be exploited to engineer metabolic pathways and thereby affect biomass recalcitrance is the simultaneous introduction, removal, and/or modification of several plant genes (i.e., gene stacking). For example, the xylan in *tbl29* mutants have a 60% reduction in *O*-acetylation, resulting in plants with reduced growth; collapsed xylem; and reduced biomass production [70]. However, overexpression of a xylan glucuronosyltransferase (GUX) enzyme in the *tbl29* mutant background functionally replaces the missing acetyl substituents with GlcA residues, restoring normal growth while maintaining low acetylation [105]. Gene-stacking approaches have also been successfully applied to increase β-1,4-galactan content in *Arabidopsis* [106]. Similar approaches to produce altered xylan structures through gene stacking, combined with the use of specific genetic regulatory elements, are an exciting and promising technique to generate novel xylan modifications with major impacts on plant recalcitrance.

In this context, one strategy to affect recalcitrance is to identify genetic modifications that change the abundance or distribution of xylan sidechain decorations in ways that modulate the strength or extent of the xylan’s interactions with itself or other cell wall polysaccharides. It has been suggested that xylan–cellulose interactions rely heavily on the presence of the major and minor domains of xylan as dictated by spacing of (Me)GlcA residues. One could imagine that altered expressions of enzymes involved in the addition of xylan substituents, including glucuronosyltransferases, α-arabinosyltransferases, β-xylosyltransferases, 4-*O*-methyltransferases, and *O*-acetyltransferases, may affect the patterning of xylan decorations in ways that disrupt polymer–polymer interactions in the wall, thereby increasing the efficiency of hydrolytic enzymes. A recent example of this idea showed how loss of the xylan acetyltransferase ESK1 results in a dysregulation of GlcA patterning, causing a loss of the normal, even spacing of GlcA sidechains and resulting in disruption of the ability of xylan to bind to cellulose fibrils [55]. Whether modifications of this type can be introduced without adversely affecting the overall wall architecture and plant fitness remains to be seen. Nevertheless, our recent work does suggest that modifying the extent of methylation of the GlcA residues is one relatively straightforward approach to increase the efficiency of biomass processing [44].

The effect of xylan on biomass recalcitrance to deconstruction is closely related to the structure and composition of the cell walls. For example, enzymatic hydrolysis of switchgrass biomass was shown to improve if xylan is previously removed from the wall by extraction with alkali, indicating that xylan is a key substrate-specific feature in switchgrass limiting sugar release [107]. The same treatment in poplar biomass is less effective, while reducing lignin content via chlorite treatment proved more beneficial [107]. Consequently, it will be necessary to find more substrate-specific approaches that address the chemical and structural differences between biomass from grasses or woody species.

Although the roles of xylan arabinosylation in grass cell wall architecture and function remain poorly understood, recent work demonstrating the xylan-specific arabinosyltransferase activities of GT61 enzymes in grasses provides new targets for xylan modification. However, perhaps the most obvious choice for modifying xylan structure to facilitate the deconstruction of grass cell walls may be to modulate the extent of feruloyl and/or coumaroyl acid substitutions. Feruloyl esters are known to crosslink cell wall polymers (especially xylans) by forming intra- and intermolecular bonds [38]. Coupling of xylan sidechains to lignin may provide strong and stable connections that impede the extraction of hemicelluloses and lignin from the wall or inhibit its enzymatic deconstruction. Increased knowledge about the enzymes responsible for the synthesis of these sidechain structures may promote genetic modifications that lead to biomass crops with more easily deconstructable walls.

## Improving biofuel production: *O*-acetylation modification

*O*-Acetylation of xylans is a key glycopolymer modification contributing to biomass recalcitrance during biofuel production. For example, acetyl groups can sterically hinder the binding of hydrolytic enzymes to their polysaccharide targets [108]. Furthermore, accumulation of acetates released during deconstruction of lignocellulosic biomass inhibits yeast growth and fermentation [109]. Regulation of xylan acetylation is a key strategy to improve biomass processing for biofuel production, and genetic engineering is a way to manipulate acetylation levels in cell wall xylans. So far, many mutants with defects in the biosynthesis of xylan acetylation have been shown to have reduced xylan acetylation levels, but they also displayed irregular xylem phenotypes and dwarfism [20, 89, 110], which is detrimental to biomass-based biofuel production. Recently, transgenic aspen lines where expression of multiple RWA genes were suppressed using a wood-specific promoter were reported to have a 25% reduction of cell wall acetylation without affecting plant growth [111]. Ground biomass from WT and reduced acetylation lines, with or without acid pretreatment, was subjected to enzymatic hydrolysis. The highest gains were observed on RWA suppression lines when enzymatic saccharification was carried out without pretreatment, resulting in 20% increased yields of all sugars per unit wood dry weight. Less pronounced effects were observed when biomass was subjected to acid pretreatment (4% increased glucose), which was likely due to removal of sugars during the pretreatment process [111].

Beyond suppressing acetylation during biosynthesis in the Golgi apparatus, expressing wall-resident xylan acetylesterases *in muro* is another strategy to optimize lignocellulosic biomass. A recent study reveals that transgenic aspen trees expressing a fungal acetyl xylan esterase had a 10% reduction in 2-*O*-monoacetylation, and an increase of cellulose crystallinity and lignin solubility. Without disturbing plant growth, these modifications increased sugar yields during enzymatic saccharification of acid pretreated biomass [112]. A similar experiment, in which a xylan acetylesterase was expressed in *Arabidopsis*, led to a 30% reduction in cell wall acetylation, and yielded 70% more ethanol relative to wild type biomass that had been pretreated with either hot water or alkali prior to fermentation [113]. Taken together, these results reinforce the notion that reducing wall acetylation increases the accessibility of hydrolytic enzymes to their polysaccharide targets in wood, which is likely due to changes in overall cell wall architecture that are imparted when the amounts and/or distribution of acetyl groups are modified.

## Conclusion

*In planta* modification of xylans remains one of the greatest challenges in feedstock bioengineering for bioindustrial purposes. This ubiquitous family of polysaccharides is composed of complex structures that can vary quite dramatically depending on species and tissue type, making further characterization of naturally occurring xylan structures an area of great interest. Recent developments have significantly furthered our knowledge about xylan synthesis and have begun to elucidate the enzymes involved in backbone elongation, sidechain addition, acetylation, and methylation. However, many areas are still black boxes waiting to be explored, including the role of reducing-end structures in xylan biosynthesis and function, enzymes responsible for the addition of ferulic/coumaric esters, precise control of chain length, and the relationships between xylan structure and its interactions with other wall components. Due to the sheer abundance of xylan in bioindustry feedstocks, it is imperative to address these gaps in biosynthetic knowledge to pave the way toward engineering better plants with less recalcitrant cell walls.

Recent advances in heterologous expression of plant cell wall GT’s in BioEnergy Science Center is finally opening the door for detailed in vitro biochemical and structural studies [64, 114], at last allowing unambiguous conclusions regarding the specific functions of proteins involved in xylan biosynthesis. This is an important step in the study of xylan biosynthesis, where many of the proteins remain uncharacterized, and the majority of knowledge concerning them has been gained solely from mutant analysis where the complexities of biology may present bewildering results. Furthermore, new insights into xylan regulation and the development of tractable genetic techniques for manipulating xylan biosynthetic machinery in tissue-specific manners will further our understanding of how gene products affect xylan structure/function in specific tissues. These results, when taken together, will provide important targets to improve biomass crops for industrial processing.

## Abbreviations

GX: glucuronoxylan
GAX: glucuronoarabinoxylan
AGX: arabinoglucuronoxylan
AX: arabinoxylan
Me: methyl
AcGX: acetylated glucuronoxylan
GT: glycosyl transferasef
IRX: irregular xylem
UXS: UDP-xylose synthase
UXT: UDP-xylose transporters
UUAT: UDP-uronic acid transporter
UXE: UDP-xylose 4-epimerase
UGF: UDP-glucose 4-epimerase
RGP: reversibly glycosylated polypeptide
UA*f*T: UDP-arabinofuranose transporters
XYS1: xylan synthase 1
AtXYS1: *Arabidopsis thaliana* xylan synthase 1
Ao: *Asparagus officionalis*
XSC: xylan synthase complex
BiFC: bimolecular fluorescence complementation
GAUT: galacturonosyltransferase
GAG: glycosaminoglycan
GUX: xylan glucuronosyl transferase
XAT: xylan arabinosyl transferase
XAX1: xylosyl arabinosyl substitution of xylan 1
VND6: vascular related NAC domain 6
VND7: vascular related NAC domain 7
SND1: secondary wall-associated NAC domain protein
MUCI21: MUCILAGE-RELATED 21
GXMT: glucuronoxylan methyl transferase 1
DUF: domain of unknown function
UDP: uridine diphosphate
RWA: reduced wall acetylation
TBL: trichome birefringence-like
AXY9: altered xyloglucan 9
ESK1: ESKIMO1
BS1: brittle leaf sheath 1
BESC: Bioenergy Science Center
